# Advances at *Preventing Chronic Disease: Public Health Research, Practice, and Policy*

**DOI:** 10.5888/pcd10.120304

**Published:** 2013-01-03

**Authors:** Samuel F. Posner

In 2003, Drs James Marks and Lynne Wilcox began developing a plan to realize their vision of creating a scholarly journal that would strengthen the links connecting public health research, practice, and policy with regard to chronic diseases. Their vision resulted in the publication the following year of *Preventing Chronic Disease: Public Health Research, Practice, and Policy* (*PCD*). As we start our 10th year of publication, we can reflect on what has been accomplished and what might be on the horizon. In the accompanying editorial, Dr Wilcox describes the importance of the journal’s focus on chronic disease in 2004 and highlights some of the early milestones in the journal’s development (1). One of the core drivers for starting *PCD* was the recognition that the field of public health would benefit from an open access forum for sharing successes and lessons learned in order to increase the quality, relevance, and effectiveness of our efforts and to improve their efficiency. The journal’s focus on chronic conditions and its commitment to providing a forum for sharing best practices and innovations is just as important today as it was a decade ago, and will increasingly be so in the future.

*PCD* has grown and changed with advances in technology and public health science to meet the changing demands of the field. In 2004, the journal was among the first to be published only in electronic format. The landscape of e-publishing has dramatically changed since then. In a world of increasing demand and constricting resources, there is an increasing push to use these resources effectively and efficiently. Since its inception, the journal has evolved to meet these challenges. *PCD* will continue to build on its solid foundation and expand its efforts by increasing its quality, relevance, impact, and efficiency.

The changes and developments at the journal in 2012 demonstrate our continued commitment to the field by incorporating the best of science, practice, policy, and publishing technology in our publication processes and content. Some of these innovations are more visible to the reader than others; however, all affect our ability to be of service to the field. First, we have increased our efficiency by redesigning our production workflow, incorporating new software that facilitates document formatting on the journal’s Web pages. Second, accompanying this change in production schedules, we can now make *PCD* articles available as soon as they are produced, without holding them to be bundled into an issue. These 2 changes have dramatically reduced the amount of time from acceptance to publication, a metric important to authors who want to see their work published more quickly and to the field to speed the process of translating research and experience into practice.

In addition to these publishing innovations, we have implemented technological innovations to increase our reach, access, and efficiency of distribution. *PCD* content is now available on multiple technology platforms through the development of the Centers for Disease Control and Prevention’s iPad application and the *PCD*-specific iPad application, both of which are available through the iTunes store under the Medical category (https://itunes.apple.com/us/app/preventing-chronic-disease/id560556547?mt=8). Additionally, the *PCD-*specific application is available for the iPhone. Journal content is also available through the CDC Content Syndication service, allowing our content to be easily placed on external websites (http://www.cdc.gov/pcd/subscriptions/syndication.htm). These advancements increase the reach of *PCD*, as do the increased distribution of summaries and content to media outlets, Facebook, and Twitter, and the continued promotion of the RSS feed; for example, our monthly Web views went from approximately 102,000 to 160,000 from January to June of 2012.

The recognition and stature of *PCD* have increased steadily since the first issue in 2004. The journal’s commitment to excellence in content and delivery is evidenced by the recognition of a growing number of ranking organizations and indexing services. Ratings of *PCD*’s impact, according to SCImago Journal Rank indicators (2), document a continual increase in our ranking from 2004 through 2010, the most recent year with complete data available. *PCD* is now indexed by a number of services including PubMed, PubMed Central, Web of Science, EBSCOhost, PsycINFO, and DOAJ (Directory of Open Access Journals), with full text content available in many of these systems. In 2012 *PCD* received a Thomson Reuters impact factor, debuting with a score of 1.819, which placed the journal in the top half of public health and environmental journals. We have also made efforts to increase *PCD*’s reach through offering continuing medical education credits, thanks to our partnership with Medscape. The quantitative data on journal ranking and use of the educational offerings are strong demonstrations of our relevance and impact in the field. If past experience is any indication of future performance, *PCD* will continue to achieve its mission of promoting an open exchange of information for all those working to reduce the burden of chronic diseases and improve health.

Publishing *PCD* is a community effort that involves researchers, reviewers, readers, and staff. Over the past several years the quality of submissions has consistently increased, as has the number, by 20%. This increase reflects the increased reach and impact of the journal in the field. In addition to contributing authors, our volunteer peer reviewers are critical to our success. The 46,000 subscribers and other readers who use the results of these efforts to implement change and improve the health of the population are critical to the success of the journal. With the guidance of the editorial board and dedication of the staff, *PCD* continues to develop from the vision of Drs Marks and Wilcox and to reflect the state of public health programs, policy, and research. It is rewarding to work with the *PCD* community, which is dedicated to efficiency in publishing processes and technology and to making the latest in evidence-based practice and practice-based evidence available to all who are working to prevent and minimize the burden of chronic diseases.

The accomplishments in 2012 exemplify *PCD’s* commitment to serving the field and provide a firm foundation for development over the next 10 years and beyond. The field of chronic disease prevention and health promotion is becoming more complex as more people have multiple chronic conditions (both physical and mental) and the once seemingly clear distinction between communicable and noncommunicable diseases becomes blurred. Furthermore, the burden of chronic diseases is increasing globally, and *PCD* is committed to being of service to researchers, practitioners, and policy makers around the world. Thank you to all who contribute to, review, read, and produce *PCD* for continuing to move the journal forward.
